# Revision of the genus *Amphirhachis* Townes, 1970 (Hymenoptera, Ichneumonidae, Banchinae) from Japan

**DOI:** 10.3897/zookeys.685.13552

**Published:** 2017-07-13

**Authors:** Kyohei Watanabe

**Affiliations:** 1 Kanagawa Prefectural Museum of Natural History, Iriuda 499, Odawara, Kanagawa 250–0031, Japan

**Keywords:** Atrophini, Far East Asia, new species, parasitoid, taxonomy

## Abstract

The Japanese species of the genus *Amphirhachis* Townes, 1970 are revised. Four species are found from Japan and two them, *A. fujiei* **sp. n.** and *A. miyabi* **sp. n.** are here described as new. A key to world species of this genus is provided.

## Introduction

The genus *Amphirhachis* Townes, 1970, is a small-sized genus of the tribe Atrophini (subfamily Banchinae), consisting of five species from the Eastern Palaearctic and Oriental regions (Yu et al. 2012, 2016). To the best of my knowledge, little is known of the biology of this genus. The species might be koinobiont endoparasitoids of lepidopterous larvae, similar to other genera of Atrophini.

In Japan, two species have been recorded, i.e. *A. nigra* Townes, 1970, from Honshu and *A. tertia* (Momoi, 1970) from Amamioshima Island and Tsushima Island (Townes 1970, Watanabeand Maeto 2012). I found some specimens of an unknown species, including many males, during a recent investigation. In the present study, I revise the Japanese species of *Amphirhachis*.

## Materials and methods

Materials used were from the collections of

**KPMNH** Kanagawa Prefectural Museum of Natural History, Odawara, Japan

**MNHAH** Museum of Nature and Human Activities, Hyogo, Sanda, Japan

**MU** Laboratory of Entomology, Meijyo University, Nagoya, Japan

**NIAES** National Institute of Agro-Environmental Sciences, Tsukuba, Japan

**SEHU** Laboratory of Systematic Entomology, Hokkaido University, Sapporo, Japan

**AEIC** American Entomological Institute, Logan, Utah, USA

**ZISP** Zoological Institute, Russian Academy of Sciences, St. Petersburg, Russia

The holotype and paratype of *A. fasciata* Chandra & Gupta, 1977, deposited in Swedish Museum of Natural History, Sweden, and a paratype of *A. rubriventris* Chandra & Gupta, 1977, deposited in AEIC were also examined. *Fintona nigripalpis* Cameron, 1909, was classified as *Amphirhachis* by a recent database (Yu et al. 2016), although I did not examine the specimen of that species. Thus I referred the character states of this species based on Chandra and Gupta (1977).

A stereomicroscope (Nikon S800) was used for observations. Photographs were taken by OLYMPUS TG-4 digital camera joined with the stereo microscope. Digital images were edited using Adobe Photoshop® CS6.

Morphological terminology mainly follows that established by Gauld (1991) and Gauld et al. (2002). Eady (1968) is referred for microsculpture description. The following abbreviations are used in the descriptions: **BWM** Basal mandibular width, **MSL** Length of malar space, **F** Segment of flagellum, **OOL** Ocello-ocular line, **POL** Postocellar line, **TS** Tarsal segment, **T** Metasomal tegite and **HT** Holotype.

The following abbreviations are used in the material data and distribution: **F** Female, **M** Male, **LT** Light trap (or came to light), **FIT** Flight interception trap, **MsT** Malaise trap and * New distributional record.

## Results

The Japanese *Amphirhachis* are classified into four species. Two of them, *A. nigra* and *A. tertia*, have been previously recorded, whereas the remaining ones, which are novel species, *A. fujiei* sp. n. and *A. miyabi* sp. n., are described below. In addition, the male of *A. nigra* is described and illustrated for the first time. All the specimens could be clearly distinguished based on morphological characters (see the key below).

### Taxonomy

#### Subfamily Banchinae Wesmael, 1845

##### Tribe Atrophini Seyrig, 1932

###### Genus Amphirhachis

Taxon classificationAnimaliaHymenopteraIchneumonidae

Townes, 1970

Amphirhachis Townes, 1970: 33. Type species: Amphirhachis nigra Townes, 1970. Original designation.

####### Description.

Body covered with short setae. Head and mesosoma mat, covered with dense punctures. Ventral margin of clypeus blunt, without a median notch. Occipital carina complete. Lower end of occipital carina connected to hypostomal carina distant from base of mandible. Antenna with tyloid-like carina on ventral surface of basal 9–14 segments in female. Mesoscutum without a raised anterolateral area on each side. Epomia absent. Epicnemial carina present laterally and ventrally. Hind rim of metanotum without a sublateral triangular projection. Fore wing with: junction of vein *Cu1* and vein *Cu-a* slightly distant or opposite to junction of *Rs+M* and *M+Cu*; *Cu-a* more or less inclivous; large areolet, receiving vein 2*m-cu* near or at its outer corner; 2*m-cu* with a single narrow bulla that is ca. 0.2 as wide as the portion of 2*m-cu* behind the bulla. Hind wing with distal abscissa of *Cu*1 meeting cu-a slightly closer to 1*A* than *M.* Tarsal claws pectinate. Posterior transverse carina of propodeum largely absent, only represented by a weak or faint vertical ridge at apex of each side and/or on median part. T1 with basal half more or less stout, its spiracle in front of middle. Median dorsal and dorsolateral carinae of T1 absent. Ovipositor sheath shorter than 0.6 times as long as hind tibia. Subgenital plate pentagonal, posterior margin weakly concave medially. Apex of paramere not projected beyond apex of aedeagus, apical margin round. Basal apodeme of aedeagus 0.3–0.4 times of total length of aedeagus.

####### Distribution.

Eastern Palaearctic and Oriental regions.

####### Remarks.

The above description is partly referred from diagnosis provided by Townes (1970), Chandra and Gupta (1973) and Kuslitzky (1995). The world species of *Amphirhachis* can be identified by the key presented below. The antennal tyloid-like carina was recognized in all Japanese species and was not found in other genera of Japanese Atrophini. These points support the hypothesis proposed by Kuslitzky (1995), i.e., that carina is one of the generic characters of *Amphirhachis*. In all Japanese species, the number of segments with that carina are varied between basal 9 to 14 segments. This number is usually 12 and the apex of the carina is never surpassed at apex of white band.

####### Key to World species of the genus *Amphirhachis*

**Table:** 

1	Base of T1 with a conspicuous yellow area or spots (Figs 16, 18, 19). Hind femur largely reddish brown (Figs 16, 19)	**2**
–	Base of T1 without yellow area or spots (Figs 1, 3, 4, 6, 9, 10, 12, 13, 15). Hind femur various in colouration	**4**
2	Base of T1 with a pair of lateral yellow spots. Posterior segments of metasoma reddish brown. Antenna with 52 flagellomeres. Scutellum black with two yellow spots. Ovipositor sheath 0.4 times as long as hind tibia. Male unknown. Myanmar and India	*A. rubriventris* Chandra & Gupta, 1977
–	Base of T1 with a wide yellow area or if with a pair of lateral yellow spots, propodeum with four yellow spots anteriorly and posteriorly (Fig. 21). Posterior segments of metasoma black and white (Figs 16, 18, 19, 21). Antenna with 47–49 flagellomeres. Scutellum entirely yellow (Figs 18, 21). Ovipositor sheath 0.4–0.5 times as long as hind tibia	**3**
3	Propodeum with four yellow spots anteriorly and posteriorly in both sexes (Figs 18, 21). Mesopleuron with two (in female, Fig. 16) or one (in male, Fig. 19) large yellow spot(s). MSL 0.5–0.6 (in female) and 0.4 (in male) times as long as BWM. T1 2.0–2.2 times as long as maximum width. Ovipositor sheath 0.4 times as long as hind tibia. Japan, Far East Russia and Kazakhstan	***A. tertia* (Momoi, 1970)**
–	Propodeum with two posterior yellow spots. Mesopleuron without two large yellow spots in female. MSL 0.7 times as long as BWM. T1 2.3 times as long as maximum width. Ovipositor sheath 0.4–0.5 times as long as hind tibia. Male unknown. Myanmar	***A. fasciatus* Chandra & Gupta, 1977**
4	T2-T7 entirely red. T1 ca. 2.0 times as long as maximum width	***A. nigripalpis* (Cameron, 1909)**
–	T2-T7 black with or without white band(s), without red area. T1 various in length	**5**
5	Body large and elongate. T1 2.2–2.8 times as long as maximum width. T2 1.1–1.3 times as long as maximum width (Figs 12, 15). Face black, usually with a pair of yellow stripes or spot(s) along inner orbit in female (Fig. 11), or yellow with a median longitudinal black stripe in male (Fig. 14). Antenna with 54–58 flagellomeres. Metasomal tergites with a posterior white band on some segments in female (Fig. 12). Ovipositor sheath 0.3–0.4 times as long as hind tibia. Japan and Far East Russia	***A. nigra* Townes, 1970**
–	Body small and not elongate. T1 1.8–2.1 times as long as maximum width. T2 0.9–1.0 times as long as maximum width (Figs 3, 6, 9). Face sometimes without a pair of yellow stripes along inner orbit in female (Fig. 8), or entirely yellow in male (Fig. 5). Antenna with 45–50 flagellomeres. Metasomal tergites with (Fig. 9) or without (Fig. 3) a posterior white band in female. Ovipositor various in length	**6**
6	Ovipositor sheath 0.3 times as long as hind tibia. Clypeus 0.5 times as long as wide. Face 0.6 times as long as wide, without a narrow longitudinal depression between eye and antennal socket (Figs 2, 5). Hind femur 5.7–6.1 times as long as maximum depth in lateral view, black. Metasomal tergite nearly entirely black in female (Figs 1, 3). Japan	***A. fujiei* sp. n.**
–	Ovipositor sheath 0.4–0.5 times as long as hind tibia. Clypeus 0.4 times as long as wide. Face 0.5 times as long as wide, with a narrow longitudinal depression between eye and antennal socket (Fig. 8). Hind femur 6.4–6.9 times as long as maximum depth in lateral view, black or reddish brown. Each metasomal tergite with a posterior white band (Figs 7, 9). Male unknown. Japan	***A. miyabi* sp. n.**

###### Amphirhachis fujiei sp. n.

Taxon classificationAnimaliaHymenopteraIchneumonidae

http://zoobank.org/47BB2E0E-EEB6-4946-816E-954337D32012

Figs 1–3 , 4–6

####### Diagnosis.

Clypeus 0.5 times as long as wide. Face 0.6 times as long as wide, without a narrow longitudinal depression between eye and antennal socket. Antenna with 45–47 flagellomeres. Hind femur 5.7–6.1 times as long as maximum depth in lateral view, black. T1 1.8–1.9 times as long as maximum width. Base of T1 without yellow area or spots. T2 0.9–1.0 times as long as maximum width. T2-T7 black with or without white band(s), without red area. Ovipositor sheath 0.3 times as long as hind tibia. Face sometimes without a pair of yellow stripes along inner orbit in female, or entirely yellow in male. Metasomal tergite nearly entirely black in female.

####### Description.

**Female** (n = 18). Body length 7.5–11.0 (HT: 9.0) mm.

*Head* 0.6 times as long as wide. Clypeus 0.5 times as long as wide. Face slightly convex medially, 0.6 times as long as wide, without a narrow longitudinal depression between eye and antennal socket (Fig. 2). Frons without a longitudinal area before anterior ocellus without punctures. POL 1.0 times as long as OOL. MSL 0.6–0.7 (HT: 0.7) times as long as BWM. Antenna with 45–47 (HT: 45) flagellomeres. F1 1.5–1.8 (HT: 1.8) times as long as F2.

*Mesosoma*. Mesopleuron without a speculum. Pleural carina present anteriorly, absent posteriorly. Fore wing length 8.0–9.5 (HT: 8.5) mm. Hind femur 5.7–6.1 (HT: 6.0) times as long as maximum depth in lateral view. Hind TS1 2.0 times as long as TS2.

*Metasoma*. T1 1.8–1.9 (HT: 1.8) times as long as maximum width. T2 0.9–1.0 (HT: 0.95) times as long as maximum width. Ovipositor sheath 0.3 times as long as hind tibia.

**Figures 1–3.**
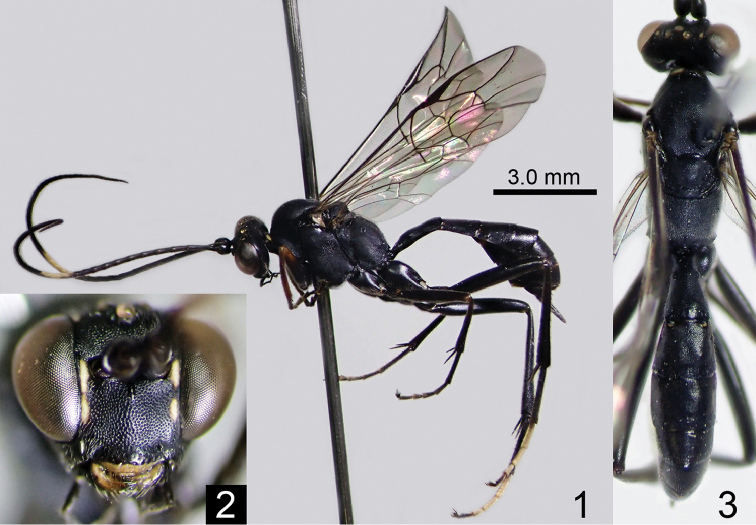
*Amphirhachis fujiei* sp. n., female (holotype). **1** lateral habitus **2** head, anterior view **3** head, mesosoma and metasoma, dorsal view.

*Colouration* (Figs 1–3). Body (excluding wings and legs) black, except for: ventral part of clypeus, a pair of spots along inner orbit of face, vertex with a pair of spots between lateral ocellus and eye, mandible excluding apex and base, a median band of flagellum, and a pair of median spots of collar white to whitish yellow. The spot of face sometimes elongated along orbit. Wings hyaline; veins and pterostigma blackish brown to brown except for yellow wing base. Legs black except for: fore and mid femora, tibiae and tarsi partly blackish brown to reddish brown and hind TS2-TS4 white to whitish yellow.

**Figures 4–6.**
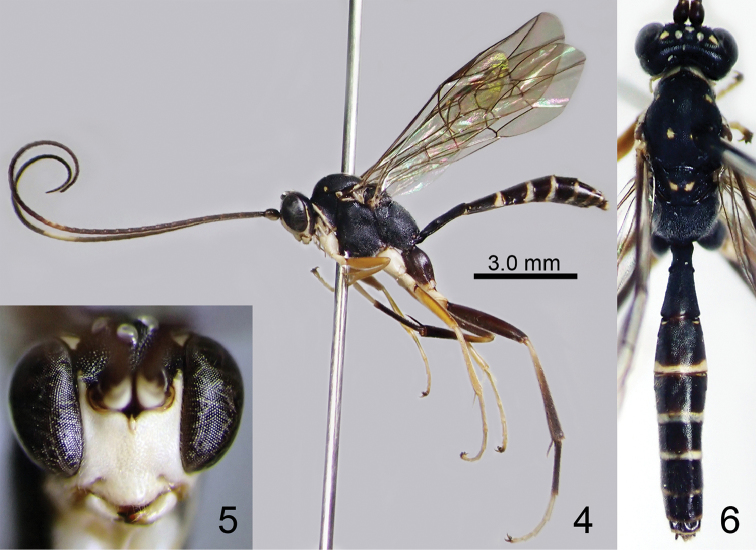
*Amphirhachis fujiei* sp. n., male (paratype). **4** lateral habitus **5** head, anterior view **6** head, mesosoma and metasoma, dorsal view.

**Male** (n = 9). In body structure, similar to female except for: POL 1.1–1.4 times as long as OOL, MSL 0.5–0.6 times as long as BWM, antenna with 45-50 flagellomeres, F1 1.4–1.6 times as long as F2, hind femur 5.4–6.2 times as long as maximum depth in lateral view, hind TS1 1.9–2.0 times as long as TS2, and T1 1.9–2.1 times as long as maximum width. In colouration, similar to the pattern of female but largely differed (Figs 4–6). Clypeus, face and malar space entirely yellow. Vertex with a pair of yellow spots between lateral ocellus and eye. Palpi, ventral spot of scape and pedicel, median band of flagellum yellow. Collar, posterodorsal corner of pronotum, subalar prominence, dorsal area of mesepimeron and posterior transverse stripe of T2-T7 whitish yellow to yellow. Mesoscutum sometimes with a pair of yellow anterolateral spots. Scutellum sometimes with a pair of yellow spots. Fore and mid legs whitish yellow to reddish yellow. Hind leg black to blackish brown except for whitish yellow trochanter and trochantellus, basal yellowish area of tibia, and TS2-TS5.

####### Specimens examined.

JAPAN: [Holotype] F, Wakayama Pref., Tanabe City, Ryujin Vil., Ryujin, 23. VI. 2012, S. Fujie leg. (KPMNH ). [Paratypes] 1 F, Hokkaido, Sapporo City, Mt. Soranuma-dake, 14. VI. – 4. VII. 2007, A. Ueda leg. (MsT) (KPMNH ); 1 M, Hokkaido, Kuriyama Town, 31. VIII. – 13. IX. 2007, A. Ueda leg. (MsT) (KPMNH ); 1 M, Hokkaido, Yubari City, Oyubari, 31. VIII. – 13. IX. 2007, A. Ueda leg. (MsT) (KPMNH ); 1 M, Hokkaido, Horikanai Town, Moshiri, Uryu, 11–17. VII. 2012, K. Watanabe et al. leg. (MsT) (KPMNH ); 1 M, Hokkaido, Mt. Tarumae-san, 12–21. VII. 1998, K. Konishi leg. (MsT.) (NIAES ); 1 F, Ibaraki Pref., Kitaibaraki, Ogawa, 27. VI. – 9. VII. 1996, K. Maeto leg. (MsT) (NIAES ); 2 F, same locality and collector, 9–25. IX. 1996 (MsT) (NIAES ); 1 M, same locality and collector, 20. VIII. – 4. IX. 1996 (MsT) (NIAES ); 3 M, Tochigi Pref., Yaita, 30. VI. – 15. VII. 1989, K. Konishi leg. (MsT) (NIAES ); 1 F, Kanagawa Pref., Odawara, 4. VI. 1987, H. Makihara leg. (NIAES ); 1 F, Kanagawa Pref., Mt. Tanzawa-san, 6. VI. 2013, T. Taniwaki leg. (FIT) (KPMNH ); 1 F, Kanagawa Pref., Mt. Oomuro-yama, 16. VI. 2013, T. Taniwaki leg. (FIT) (KPMNH ); 1 F, Kanagawa Pref., Mt. Komotsurushi-yama, 21. VI. 2013, T. Taniwaki leg. (FIT) (KPMNH ); 1 M, Yamanashi Pref., Hokuto City, Masutomi, Biwakubo-sawa, 28. VII. 2007, T. Ban leg. (KPMNH ); 1 F, Yamanashi Pref., Tsuru City, Matsuhime-toge, 28. VII. – 9. VIII. 2008, T. Sakurai & T. Zakoji leg. (KPMNH ); 1 F, Yamanashi Pref., Koushu City, Yamato Town, Tokusa, 7. VIII. 2008, T. Muraki leg. (KPMNH ); 1 F, Nagano Pref., Shimashima-dani, 28. VII. 1980, H. Takemoto leg. (NIAES ); 1 F, Nagano Pref., Outaki Vil., Mt. Ontake-san, Hakkaisan, 8–9. VIII. 2014, S. Shimizu leg. (MsT.) (KPMNH ); 1 F, Hyogo Pref., Kami Town, Niiya, Mikata-kogen (710 m alt.), 26. VI. – 18. VII. 2011, S. Fujie leg. (MsT) (KPMNH ); 1 F, Tottori Pref., Mt. Daisen, 24. VI. 1978, Y. Yoneda leg. (NIAES ); 1 F, Tottori Pref., Wakasa Town, Mt. Hyonosen, Oodanganaru, 6. VIII. 2011, K. Watanabe leg. (LT) (KPMNH ); 1 F, Ehime Pref., Odamiyama, 25. VII. 1995, E. Yamamoto leg. (LT) (NIAES ); 1 F, Fukuoka Pref., Mt. Hiko, 5–6. VII. 1979, K. Maeto leg. (NIAES ).

####### Distribution.

Japan (Hokkaido, Honshu, Shikoku and Kyushu).

####### Etymology.

The specific name is from my friend, Mr. Shunpei Fujie, who is a taxonomist of Japanese Braconidae and a collector of types.

####### Bionomics.

Host is unknown. The specimens collected from Hyogo (Mt. Hyonosen) and Shikoku were collected by light trap.

####### Remarks.

This species resembles *A. nigra* in the body colouration, but it can be distinguished by the T1 1.8–2.1 times as long as maximum width (2.2–2.8 times in *A. nigra*), T2 0.9–1.0 times as long as maximum width (1.1–1.3 times in *A. nigra*), the body length 7.0–7.5 mm (54–58 flagellomeres in *A. nigra*) and T2 without a posterior white band in female (usually with a white band in female of *A. nigra*). Males were collected only from Hokkaido and Honshu.

###### Amphirhachis miyabi sp. n.

Taxon classificationAnimaliaHymenopteraIchneumonidae

http://zoobank.org/CEDF0413-1954-4DCE-A9C6-353DB0CB7A68

Figs 7–9

####### Diagnosis.

Clypeus 0.4 times as long as wide. Face 0.5 times as long as wide, with a narrow longitudinal depression between eye and antennal socket. Antenna with 46–49 flagellomeres. Hind femur 6.4–6.9 times as long as maximum depth in lateral view, black or reddish brown. T1 1.9–2.0 times as long as maximum width. Base of T1 without yellow area or spots. T2 0.9–1.0 times as long as maximum width. Each metasomal tergite with a posterior white band. Ovipositor sheath 0.4–0.5 times as long as hind tibia.

####### Description.

**Female** (n = 18). Body length 10.0–11.0 (HT: 10.0) mm.

*Head* 0.6 times as long as wide. Clypeus 0.4 times as long as wide. Face slightly convex medially, 0.5 times as long as wide, with a narrow longitudinal depression between eye and antennal socket (Fig. 8). Frons with a longitudinal area before anterior ocellus without punctures. POL 0.8–1.0 (HT: 0.8) times as long as OOL. MSL 0.7–0.8 (HT: 0.7) times as long as BWM. Antenna with 46–49 (HT: 46) flagellomeres. F1 1.6–1.7 (HT: 1.6) times as long as F2.

*Mesosoma*. Mesopleuron with a very small speculum. Pleural carina present but trace-like in entire length. Fore wing length 9.0–10.0 (HT: 9.0) mm. Hind femur 6.4–6.9 (HT: 6.9) times as long as maximum depth in lateral view. Hind TS1 2.0–2.1 (HT: 2.1) times as long as TS2.

*Metasoma*. T1 1.9–2.0 (HT: 1.9) times as long as maximum width. T2 0.9–1.0 (HT: 0.9) times as long as maximum width. Ovipositor sheath 0.4–0.5 (HT: 0.5) times as long as hind tibia.

**Figures 7–9.**
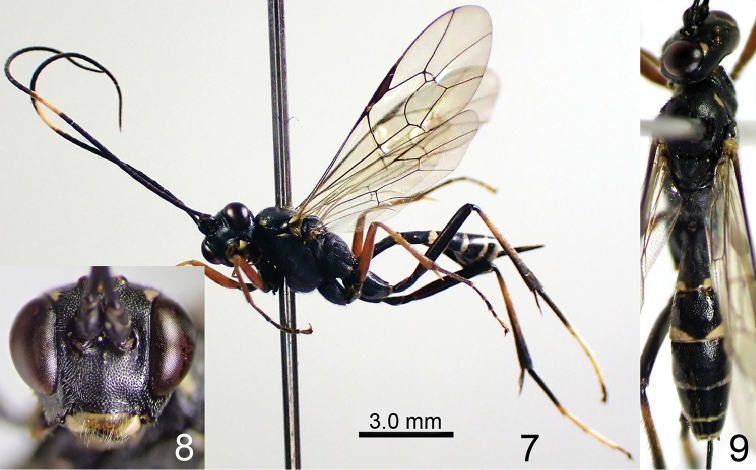
*Amphirhachis miyabi* sp. n., female (holotype). **7** lateral habitus **8** head, anterior view **9** head, mesosoma and metasoma, dorsal view.

*Colouration* (Figs 7–9). Body (excluding wings and legs) black, except for: clypeus excluding dorsal part, vertex with a pair of spots between lateral ocellus and eye, mandible excluding apex and base, a median band of flagellum, anterolateral spots of mesoscutum, subalar prominence, apical spots of T1, apical transverse stripe of T2-T7 white to whitish yellow; palpi brown; metasomal sternites blackish brown brown basally, whitish brown apically. Wings hyaline; veins and pterostigma blackish brown to brown except for yellow wing base. Coxae, trochanters and trochantelli black. Fore and mid femora, tibiae and tarsi reddish brown to brown. Hind femur, base and apical part of hind tibia, basal 0.7–0.8 (HT: 0.8) of hind TS1, and TS5 black to blackish brown. Subbasal area of hind tibia reddish brown. Apical 0.2 of hind TS1 and TS2-TS4 whitish yellow. In some paratypes with: collar with yellow spot medially, mesoscutum with a indistinct, median yellow spot, scutellum with a pair of small yellow spots, and propodeum with a pair of small yellow spots near the socket of hind coxa. The apical yellow spot of T1 usually united into a single transverse stripe. In specimens collected in Yakushima Is., hind femur sometimes tinged with reddish brown. Ovipositor reddish brown.

**Male**. Unknown.

####### Specimens examined.

JAPAN: [Holotype] F, Nagasaki Pref., Tsushima Is., Kamitsushima Town, Izumi, Shitazaki (ca. 10 m alt.), 8. V. 2015, T. Kurihara leg. (KPMNH ). [Paratypes] 2 F, Aichi Pref., Nagoya City, Higashiyama Park, 1–10. V. 2001, M. Watanabe leg. (MsT) (MU); 2 F, same locality and collector, 11–20. V. 2001 (MsT) (MU); 5 F, Aichi Pref., Toyota City, Takiwaki, 30. IV. – 6. V. 2002, Y. Kurahashi leg. (MsT) (MU); 2 F, Aichi Pref., Toyota City, Sanage, 30. IV. – 6. V. 2002, M. Kiyota leg. (MsT) (MU); 1 F, Miyazaki Pref., Mt. Wanizuka, 23. V. 1966, K. Kusigemati leg. (SEHU); 1 F, Kagoshima Pref., Yakushima Is., Kosugidani, 2. VI. 1969, K. Kusigemati leg. (SEHU); 1 F, same locality and collector, 3. VI. 1969 (SEHU); 3 F, Kagoshima Pref., Yakushima Is., Shiratani (600 m alt.), 6. V. – 20. VI. 2000, T. Murata leg. (MsT) (MU).

####### Distribution.

Japan (Honshu, Kyushu, Tsushima Is. and Yakushima Is.),

####### Etymology.

The specific name is from a Japanese term “Miyabi”, which means elegant.

####### Bionomics.

Host is unknown.

####### Remarks.

This species resembles *A. fujiei* sp. n. in the body structures, but it can be distinguished by ovipositor sheath 0.4–0.5 times as long as hind tibia (0.3 times in *A. fujiei* sp. n.) and the each metasomal tergite with a posterior white band (entirely black in female of *A. fujiei* sp. n.).

###### Amphirhachis nigra

Taxon classificationAnimaliaHymenopteraIchneumonidae

Townes, 1970

Figs 10–12 , 13–15

Amphirhachis nigra Townes, 1970: 33.

####### Description.

**Female** (n = 32). Body length 10.0–12.5 mm.

*Head* 0.6 times as long as wide. Clypeus 0.5 times as long as wide. Face slightly convex medially, 0.6 times as long as wide, without a narrow longitudinal depression between eye and antennal socket (Fig. 11). Frons densely punctate with rugae above each antennal socket, without a longitudinal area before anterior ocellus without punctures. POL 0.8–1.0 times as long as OOL. MSL 0.7 times as long as BWM. Antenna with 56-57 flagellomeres. F1 1.5–1.6 times as long as F2.

*Mesosoma*. Mesopleuron without a speculum. Pleural carina present but trace-like in entire length. Fore wing length 9.0–12.5 mm. Hind femur 7.1–8.1 times as long as maximum depth in lateral view. Hind TS1 1.9–2.0 times as long as TS2.

*Metasoma*. T1 2.2–2.8 (usually 2.2–2.3) times as long as maximum width. T2 1.1–1.3 times as long as maximum width. Ovipositor sheath 0.3–0.4 (usually 0.3) times as long as hind tibia.

**Figures 10–12.**
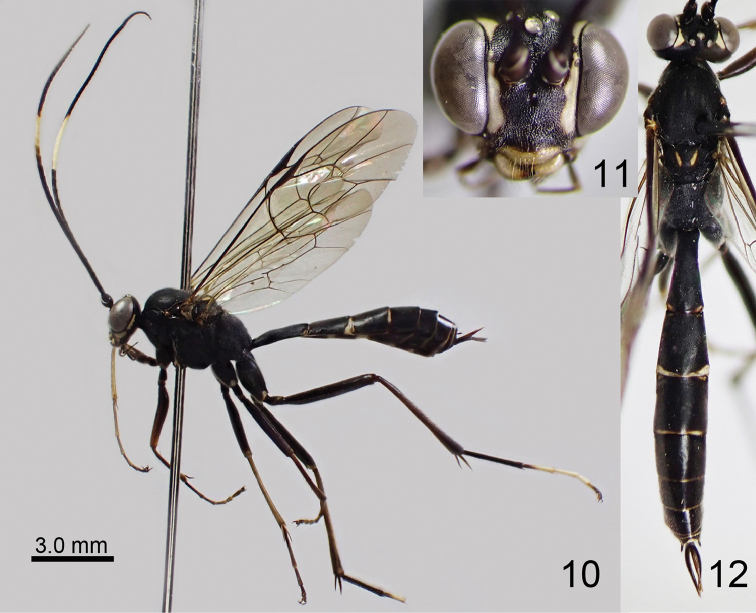
*Amphirhachis nigra* Townes, 1970, female from Japan. **10** lateral habitus **11** head, anterior view **12** head, mesosoma and metasoma, dorsal view.

*Colouration* (Figs 10–12). Body (excluding wings and legs) black, except for: clypeus except for dorsal part, longitudinal stripes along orbit except for ventral part of face and dorsal part of gena, mandible excluding apex and base, a median band of flagellum, a pair of median spot of collar, a pair of small spot of scutellum, anterolateral small spots of T1, posterior transverse stripe of T2, narrow posterior margin of T3-T6, base and apex of S1, posterior transverse stripe of S2-S6 whitish yellow; palpi, ventral surface of scape and pedicel, posterodorsal corner of pronotum, subalar prominence, and dorsal area of mesepimeron tinged with brown to reddish brown. The marking along orbit sometimes partly reduced. Scutellum sometimes without yellow markings. Stripes of metasomal tergites sometimes reduced but usually present on T2. Wings hyaline; veins and pterostigma blackish brown to brown except for yellow wing base. Legs black except for: fore and mid femora, tibiae and tarsi partly blackish brown to reddish brown and hind TS2-TS4 white to whitish yellow. Hind femur and tibia sometimes tinged with reddish brown. Hind TS2 sometimes black basally.

**Figures 13–15.**
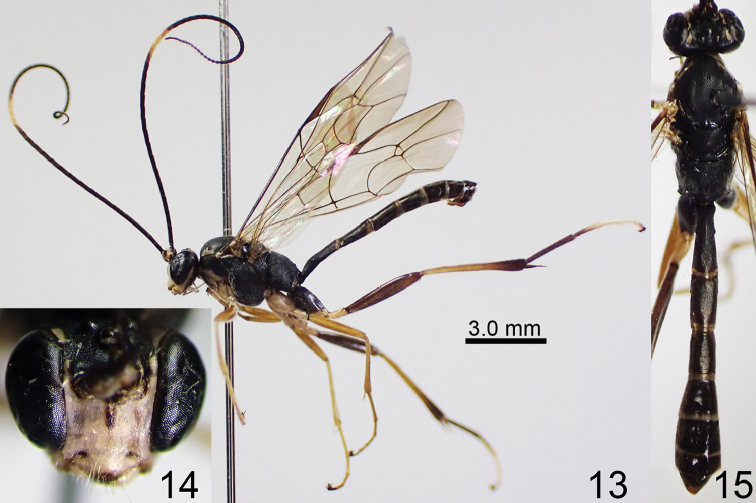
*Amphirhachis nigra* Townes, 1970, male from Japan. **13** lateral habitus **14** head, anterior view **15** head, mesosoma and metasoma, dorsal view.

**Male** (n = 24). In body structure, similar to female except for: MSL 0.4–0.6 times as long as BWM, antenna with 54-58 flagellomeres, F1 1.4–1.6 times as long as F2, anterior part of pleural carina well-developed, T1 relatively longer than female (usually 2.5–2.6 times as long as maximum width), hind femur 6.5–7.7 times as long as maximum depth in lateral view, hind TS1 1.8–1.9 times as long as TS2 and T2 1.4–1.8 times as long as maximum width. In colouration, similar to the pattern of female but largely differed (Figs 13–15). Clypeus and malar space entirely yellow. Face yellow except for median longitudinal black stripe. Palpi, ventral spot of scape and pedicel, and median band of flagellum whitish yellow to yellow. Collar, posterodorsal corner of pronotum, anterolateral spots of mesoscutum, subalar prominence, dorsal area of mesepimeron and posterior transverse stripe of T1-T7 whitish yellow to yellow. Propleuron sometimes with a whitish yellow to yellow spot. Mesoscutum sometimes with a median yellow spot. Mesopleuron usually along mesosternum with a longitudinal whitish yellow stripe. Mesepimeron sometimes entirely whitish yellow. Propodeum sometimes with a pair of small yellow spots near the socket of hind coxa. Fore and mid legs whitish yellow to reddish yellow. Hind coxa partly tinged with whitish yellow. Hind trochanter and trochantellus whitish yellow. Hind femur, tibia and tarsus more or less paler than female especially basal part of tibia largely reddish brown.

####### Specimens examined.

JAPAN: 1 M, Hokkaido, Mt. Tarumae-san, 12–21. VII. 1998, K. Konishi leg. (MsT.) (NIAES ); 1 F, same locality and collector, 21–26. VII. 1998 (MsT) (NIAES ); 8 M, Hokkaido, Sapporo City, Mt. Soranuma-dake, 14. VI. – 4. VII. 2007, A. Ueda leg. (MsT) (KPMNH ); 1 F, same locality and collector, 27. VII. – 21. VIII. 2007 (MsT) (KPMNH ); 2 M, Hokkaido, Hidaka Town, Uenzaru-gawa, 21. VI. – 10. VII. 2007, A. Ueda leg. (MsT) (KPMNH ); 1 M, same locality and collector, 10. VII. – 1. VIII. 2007 (MsT) (KPMNH ); 1 M, Hokkaido, Eniwa City, Izari, Ichankoppe-zawa, 20–30. VI. 1995, T. Ito leg. (MsT) (NIAES ); 1 M, same locality and collector, 12–20. VII. 1995 (MsT) (NIAES ); 1 F, same locality and collector, 21–31. VII. 1995 (MsT) (NIAES ); 1 M, Kanagawa Pref., Atsugi City, Mt. Oyama (1250 m alt.), 7. VI. 1997, M. Kato leg. (KPMNH ); 1 F, Kanagawa Pref., Mt. Tanzawa-san, 16. V. 2013, T. Taniwaki leg. (FIT) (KPMNH ); 1 M, same locality and collector, 20. VI. 2013 (FIT) (KPMNH ); 1 M, Kanagawa Pref., Mt. Oomuro-yama, 16. VI. 2013, T. Taniwaki leg. (FIT) (KPMNH ); 2 M, Kanagawa Pref., Mt. Hinokiboramaru, 23. V. 2013, T. Taniwaki leg. (FIT) (KPMNH ); 2 M, Kanagawa Pref., Mt. Hinokiboramaru, 6. VI. 2013, T. Taniwaki leg. (FIT) (KPMNH ); 1 F, Shizuoka Pref., Kanaya City, Fukuyo, 16. XI. 1952, J. Minamikawa leg. (NIAES ); 1 F, Shizuoka Pref., Kanaya, 1. XII. 1955, J. Minamikawa leg. (NIAES ); 1 F (holotype), Nagano Pref., Kamikochi, 31. VII. 1954, Townes family leg. (AEIC); 1 F, Nagano Pref., Mt. Shiroumayari, 29. VII. 1972, T. Aoki leg. (NIAES ); 1 F, Nagano Pref., Outaki Vil., Mt. Ontake-san, Hakkaisan, 16. IX. 2011, S. Fujie leg. (KPMNH ); 1 M, same locality, 8–9. VIII. 2014, S. Shimizu leg. (MsT.) (KPMNH ); 1 M, Ishikawa Pref., Mt. Hakusan, 5. VIII. 1993, I. Togashi leg. (NIAES ); 1 M, same locality and collector, 8. IX. 1996 (NIAES ); 1 F, Mie Pref., Owase, 16. XI. 1958, S. Ishida leg. (NIAES ); 1 F, “Odaigahara”, 6. VI. 2004, A. Kawazoe leg. (KPMNH ); 1 F, Nara Pref., Odaigahara (1500 m alt.), 25. VI. 1984, K. Konishi leg. (NIAES ); 1 F, Ehime Pref., Odamiyama, 15. VI. 1990, E. Yamamoto leg. (NIAES ); 1 F, same locality and collector, 13. V. 1994 (NIAES ); 1 F, same locality and collector, 27. V. 1994 (NIAES ); 1 F, same locality and collector, 28. V. 1994 (NIAES ); 1 F, same locality and collector, 26. VI. 1994 (NIAES ); 2 F, same locality and collector, 1. VI. 1995 (NIAES ); 1 F, same locality and collector, 5. VI. 1995 (NIAES ); 1 F, same locality and collector, 11. VI. 1995 (NIAES ); 2 F, same locality and collector, 25. VI. 1995 (NIAES ); 1 F, same locality and collector, 10. VII. 1995 (NIAES ); 1 F, Tokushima Pref., Mt. Tsurugisan, 19. VI. 1981, I. Kanazawa leg. (LT) (NIAES ); 2 F, Kumamoto Pref., Mt. Aso, 12. VI. 1978, H. Makihara leg. (LT) (NIAES ); same locality and collector, 18. VI. 1978 (LT) (NIAES ); 1 F, Kumamoto Pref., Izumi Vil., Mt. Shiratori-yama (1300 m alt.), 6. VI. 1980, K. Ohara leg. (NIAES ); 1 F, Kumamoto Pref., Izumi Vil., “Mt. Hakuchō-zan” (= Mt. Shiratori-yama ??), 14. V. 1983, K. Ohara leg. (NIAES ); 1 F, same locality, 14–21. V. 1983, K. Ohara & T. Goto leg. (MsT) (NIAES ); 1 F, Kumamoto Pref., Shiiya-toge (1400 m alt.), 15. VI. 1985, K. Konishi et al. leg. (LT) (NIAES ).

####### Distribution.

Japan (Hokkaido, Honshu, Shikoku and Kyushu); Far East Russia.

####### Bionomics.

Host is unknown. Some specimens collected from Shikoku and Kyushu were collected by light trap.

####### Remarks.

This is the first record of the male. Males were collected only from Hokkaido and Honshu.

###### Amphirhachis tertia

Taxon classificationAnimaliaHymenopteraIchneumonidae

(Momoi, 1970)

Figs 16–18 , 19–21

Fintona tertia Momoi, 1970: 375.
Amphirhachis quadripunctata Kuslitskiy, 1995: 674.

####### Description.

**Female** (n = 4). Body length 10.0–11.0 mm.

*Head* 0.6 times as long as wide. Clypeus 0.4 times as long as wide. Face slightly convex medially, 0.6–0.7 times as long as wide, without a narrow longitudinal depression between eye and antennal socket (Fig. 17). Frons densely punctate with transverse creases above each antennal socket, with a longitudinal area before anterior ocellus without punctures. POL 0.9 times as long as OOL. MSL 0.5–0.6 times as long as BWM. Antenna with 47–50 flagellomeres. F1 1.5–1.8 times as long as F2.

*Mesosoma*. Mesopleuron without speculum. Pleural carina present but trace-like in entire length. Fore wing length 8.0–8.5 mm. Hind femur 6.1–6.4 times as long as maximum depth in lateral view. Hind TS1 2.0–2.1 times as long as TS2.

Metasoma. T1 2.0–2.2 times as long as maximum width. T2 0.9–1.0 times as long as maximum width. Ovipositor sheath 0.4 times as long as hind tibia.

**Figures 16–18.**
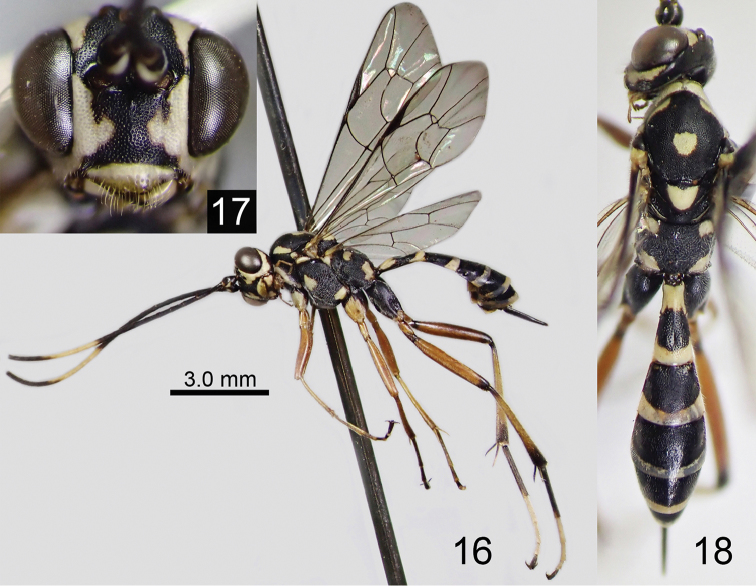
*Amphirhachis tertia* Momoi, 1970, female from Japan. **16** lateral habitus **17** head, anterior view **18** head, mesosoma and metasoma, dorsal view.

*Colouration* (Figs 16–18). Body (excluding wings and legs) black with some whitish yellow markings. The yellow area on head are: mandible except for base and apex, clypeus except for dorsal margin, stripe along orbit except for dorsal part of gena, ventral spot of scape and pedicel, and a median band of flagellum. Yellow stripe on face widened medially. The yellow area on mesosoma are: collar and posterodorsal corner of pronotum, anterolateral longitudinal spots and a median spot of mesoscutum, scutellum, subalar prominence, dorsal area of mesepimeron, two large spots on mesopleuron, four spots on propodeum. Wings hyaline; veins and pterostigma blackish brown to brown except for yellow wing base. Fore and mid coxae whitish yellow, with small black area. Fore and mid trochanters, trochanteli and tarsi yellowish brown. Fore and mid femora and tibiae reddish brown. Hind coxa, base and apex of hind femur, base and apical part of hind tibia, hind TS1 and TS5 black to blackish brown. Hind trochanter, trochantellus, femur and tibia except for black area reddish brown. Hind TS2-TS4 white. The yellow area on mesosoma are: basal spot and apical transverse stripe of T1, apical transverse stripe of T2-T7. Metasomal sternites blackish brown brown basally, whitish brown apically. Ovipositor reddish brown.

**Figures 19–21.**
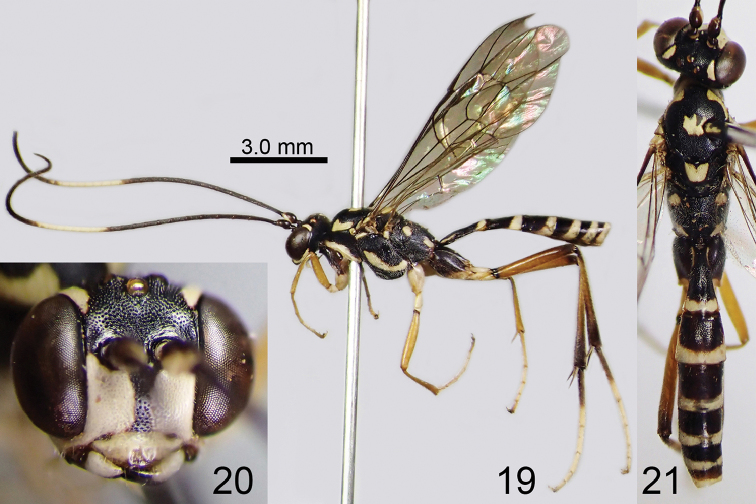
*Amphirhachis tertia* Momoi, 1970, male from Japan. **19** lateral habitus **20** head, anterior view **21** head, mesosoma and metasoma, dorsal view.

**Male**. Similar to female (Figs 19–21) except for: MSL 0.4 times as long as BWM, face largely white except blackish areas along each antennal socket and median longitudinal line, yellowish spots on mesopleuron united as transverse band, blackish areas on fore and middle coxae reduced, hind TS5 white; basal white area of first metasomal tergite reduced, and only longitudinal line between spiracle and base present.

####### Specimens examined.

JAPAN: 1 F (holotype of *Fintona tertia*), Kagoshima Pref., Amamioshima Is., 6. V. 1959, K. Kamijo leg. (MNHAH ; 1 F, Kagoshima Pref., Amamioshima Is., Mt. Yuidake, 1. IV. 1989, Y. Takematsu leg. (NIAES ); 1 F, Kagoshima Pref., Amamioshima Is., Yuwan, 29. III. 2015, Y. Fujisawa leg. (KPMNH ); 1 M, Nagasaki Pref., Tsushima Is., Mt. Mitake, 3. V. 1989, K. Konishi leg. (NIAES ). KAZAKHSTAN: 1 F (holotype of *Amphirhcachis quadripunctata*), Andreyevka, 3. VIII. 1985, S. Belokobylskij leg. (ZISP).

####### Distribution.

Japan (Tsushima Is. and Amamioshima Is.); Far East Russia (Primorye Kray) and Kazakhstan.

####### Bionomics.

Host is unknown.

####### Remarks.

The distribution data for this species is relatively sparse as compared to other species. The locality of Kazakhstan is distant from Japan and Far East Russia (Primorye Kray) while no differences of character states were found between both specimens.

## Supplementary Material

XML Treatment for Amphirhachis

XML Treatment for Amphirhachis fujiei

XML Treatment for Amphirhachis miyabi

XML Treatment for Amphirhachis nigra

XML Treatment for Amphirhachis tertia
